# Malaria infection and the risk of epilepsy: a meta-analysis

**DOI:** 10.1017/S0031182022001780

**Published:** 2023-04

**Authors:** Ali Alizadeh Khatir, Mahdi Sepidarkish, Yasaman Daryabari, Ali Taghipour, Abolfazl Mollalo, Saeed Aghapour, Ali Rostami

**Affiliations:** 1 Mobility Impairment Research Center, Health Research Institute, Babol University of Medical Sciences, Babol, Iran; 2 Department of Biostatistics and Epidemiology, School of Public Health, Babol University of Medical Sciences, Babol, Iran; 3 Infectious Diseases and Tropical Medicine Research Center, Health Research Institute, Babol University of Medical Sciences, Babol, Iran; 4 Zoonoses Research Center, Jahrom University of Medical Sciences, Jahrom, Iran; 5 Department of Public Health and Prevention Science, School of Health Sciences, Baldwin Wallace University, Berea, Ohio, USA; 6 Department of Neurosurgery, Faculty of Medicine, Mazandaran University of Medical Sciences, Sari, Iran

**Keywords:** Cerebral malaria, epilepsy, malaria infection, meta-analysis, systematic review

## Abstract

Epilepsy, a chronic disease of the central nervous system, is highly prevalent in malaria-endemic regions. Therefore, several studies have evaluated the associations between malaria infection and epilepsy development. A meta-analysis of observational studies published from inception to 10 May 2022 has been conducted to synthesize and pool the existing data on this topic. The relevant publications were systematically searched in PubMed/Medline, Scopus, Embase and Web of Science database collections. A random-effects meta-analysis model (REM) was utilized to generate the pooled odds ratio (OR) at 95% confidence intervals (CIs). The between-studies heterogeneity was assessed with *I*^2^, as well as several subgroups, meta-regression and sensitivity analysis were performed to identify the source of heterogeneity. Overall, 17 eligible studies containing 6285 cases and 13 909 healthy controls were included. The REM showed a significant positive association between malaria infection and epilepsy development (OR 2.36; 95% CI 1.44–3.88). In subgroup analyses, significant positive associations were observed in studies that: epilepsy was the outcome in the follow-up of patients with cerebral malaria (OR 7.10; 95% CI 3.50–14.38); used blood smear to diagnose malaria (OR 4.80; 95% CI 2.36–9.77); included only children (OR 3.92; 95% CI 1.81–8.50); published before 2010 (OR 6.39; 95% CI 4.25–9.62). Our findings indicated that patients with malaria, especially those with cerebral malaria, are at a high risk of epilepsy development; however, further well-designed and controlled studies are needed to verify the strength of the association.

## Graphical abstract

**Figure:**
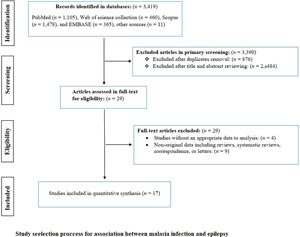

## Introduction

Epilepsy, a chronic disease of the central nervous system, is characterized by abnormal brain electrical activity leading to seizures, stereotyped behavioural alterations and occasionally loss of awareness (WHO, 2019). Epilepsy and its related consequences account for a substantial proportion of the world's burden of neurological diseases (Beghi *et al*., 2019; WHO, 2019). The global burden of disease has been estimated to be 45.9 million patients (39.9–54.6 million) with all-active epilepsy, accounting for more than 13.5 million disability-adjusted life years and 126 000 deaths in 2016 (Beghi *et al*., 2019). In developed countries, the overall incidence of epilepsy is 48.9 per 100 000 person-years. However, this proportion is 2–3 times higher in low- and middle-income countries (LMIC) (139 per 100 000 person-years) (WHO, 2019), where zoonotic and vector-borne infections are predominantly reported (Ngugi *et al*., 2013; Singh *et al*., 2020). It has been estimated that almost 80% of people with epilepsy live in LMIC, especially in sub-Saharan Africa (Beghi *et al*., 2019; WHO, 2019). The high incidence of epilepsy in these areas is attributed to some neurotropic infections, including cysticercosis, toxocariasis, onchocerciasis, toxoplasmosis and malaria (Ngugi *et al*., 2013; Singh *et al*., 2020).

Malaria is a life-threatening infection caused by *Plasmodium* parasites, transmitted to humans by the bite of the infected female Anopheles mosquito (WHO, 2020). Malaria infection is endemic in Africa, Asia and South America (WHO, 2020). The World Malaria Report has estimated 241 million malaria cases and 627 000 malaria-related deaths in 2020 worldwide, of which 95% of all malaria cases and 96% of deaths were from the WHO African region (WHO, 2020). There are 5 *Plasmodium* species causing malaria infection in humans, of which *P. vivax* (dominant outside Africa) and *P. falciparum* (dominant in Africa) are the most common species with the greatest threats (Guerra *et al*., 2006; Battle *et al*., 2019). Cerebral malaria (CM), characterized by coma and parasitaemia, is a life-threatening consequence of malaria infection, mainly induced by *P. falciparum* and rarely by *P. vivax* (Hora *et al*., 2016; Luzolo and Ngoyi, 2019; Mukhtar *et al*., 2019). CM has a case-fatality rate of 15–20% and can cause several neurological sequelae in survivors, including language regression, cortical blindness, ataxia, gross motor deficits, behavioural abnormalities, seizure and epilepsy (Brewster *et al*., 1990; Carme *et al*., 1993; van Hensbroek *et al*., 1997; Ngoungou *et al*., 2007; Birbeck *et al*., 2010).

Seizures and other status epilepticus are common neurological manifestations in children with malaria, especially those with CM. However, a significant proportion of these manifestations may be simple febrile seizures (Kariuki *et al*., 2013; Angwafor *et al*., 2019). Most seizures are prolonged with focal characteristics occurring when body temperature is less than 38°C implying that other mechanisms besides fever, probably directly related to the parasite, are involved (Waruiru *et al*., 1996; Angwafor *et al*., 2019). Despite this evidence, there are several gaps in our understanding of the relationship between malaria infection and development of epilepsy. A systematic review by Christensen and Eslick (2015) demonstrated a significant positive association between CM and epilepsy. However, the review only included studies on severe CM, necessitating a more focused study on all malaria infections. Moreover, several recent studies have been published in this area, indicating the need for an updated comprehensive meta-analysis. Therefore, to address this gap, the present study assesses the relationship between malaria infection and epilepsy development using a comprehensive meta-analysis to summarize and include updated findings.

## Materials and methods

This study follows the Preferred Reporting Items for Systematic Reviews and Meta-Analyses (PRISMA) statement as guidance to design the study and report and interpret the findings (Moher *et al*., 2015).

### Search strategy and study selection

Four literature databases were systematically searched for relevant publications, including PubMed/Medline, Scopus, Embase and Web of Science collection, from inception to 10 May 2022. The inclusive keywords composed of malaria, severe malaria, cerebral malaria, brain malaria, *Plasmodium*, *Plasmodium falciparum*, *Plasmodium vivax*, epilepsy, seizure, neurological complications, neurological sequelae, relationship and association. The search process was performed by combining these keywords using ‘OR’ and/or ‘AND’ logical operators. The detailed search strategy in databases is presented in Fig. S1. To avoid potential missing relevant publications and grey literature, other sources such as Google Scholar, OpenGrey, ProQuest and references list of all studies were searched and included in the meta-analysis. No language restrictions were applied, and articles in languages other than English were translated to English using the online tool ‘Google Translate’ (https://translate.google.com/). All retrieved publications were exported to EndNote Reference Manager X8 (Clarivate Analytics, Philadelphia, PA, USA) where duplicates were removed.

### Inclusion and exclusion criteria

After duplicate removal, 2 reviewers (A. A. K. and A. R.) independently screened the titles and abstracts of identified references, followed by full-text screening based on predefined inclusion and exclusion criteria. All the discrepancies were resolved by consulting with the lead investigator (A. R.). The main criteria for inclusion were: (1) peer-reviewed observational studies with cross-sectional, cohort or case–control design; (2) studies that included patients with confirmed malaria infection or patients with epilepsy as the cases, and the controls were individuals without malaria, or without the epilepsy/neurological complications; (3) studies that used internationally recognized diagnostic assays or guidelines to diagnose malaria infection or epilepsy; (4) studies that the risk point estimate was reported as an odds ratio (OR) and confidence intervals (CIs), or the information was presented such that an OR and 95% CI could be calculated. The exclusion criteria for this study included (1) studies that failed to quantitatively assess the relationship between malaria and epilepsy; (2) studies that an OR and 95% CI could not be calculated; (3) studies without original data (e.g. systematic reviews and letters); (4) case reports and case-series studies; (5) full-text of the article was not available (e.g. abstract or conference articles).

### Data extraction and quality assessment

Two reviewers (A. T. and A. A. K.) independently extracted the following information from each eligible study using a standardized data extraction form: first author, publication year, type of participants (malaria-based or epilepsy-based), study design (cross-sectional, cohort or case–control), type of population (adult, children or both), country, diagnostic assay for malaria infection (blood smear, serology or medical history), the number of cases and control subjects, the prevalence of epilepsy or malaria in individuals of each of the subject groups. The quality of cohort and case–control studies was assessed by the Newcastle–Ottawa Scale (NOS) (Text S1), as endorsed by the Cochrane network (Stang, 2010; Higgins *et al*., 2019). Moreover, an adapted NOS was used for the quality assessment of cross-sectional studies (Text S2) (Herzog *et al*., 2013). In both scoring scales, the quality of each eligible study was rated as high (7–9 scores), moderate (4–6 scores) or poor (0–3 scores).

### Data synthesis and statistical analysis

Meta-analyses were performed using Stata software ver. 17 (Stata Corporation, College Station, TX, USA). The pooled prevalence of epilepsy or malaria infection for each case and control group was estimated at a 95% CI using the DerSimonian–Laird random-effects model (REM) (DerSimonian and Laird, 2015). The variances in the meta-analysis were stabilized by transforming the raw prevalence estimates using the Freeman–Tukey double arcsine transformation (Hamza *et al*., 2008). The ORs from individual studies were calculated to assess the association between malaria infection and epilepsy in each study. Then, the ORs from individual studies were combined to produce a pooled OR, employing the REM with a restricted maximum-likelihood estimator. Between-study heterogeneity was evaluated by the Cochrane *Q* test and *I*^2^ statistics. The *I*^2^ value greater than 75% was considered as considerable heterogeneity (Higgins *et al*., 2003). The results were stratified into subgroups by diagnostic methods, study design, type of participants and type of studies to better explore the potential effect modification by the study characteristics on study outcome as well as potential sources of heterogeneity. The robustness of the results was evaluated by iteratively removing 1 study to assess each study's influence on the pooled estimate for both outcomes. Potential publication bias was determined using a contour-enhanced funnel plot and Egger's test, as up to 10 eligible studies were included in the meta-analysis (Egger *et al*., 1997). A cumulative meta-analysis was conducted to explore the trend of evidence accumulation. Results with *P* < 0.05 (2-sided) were considered statistically significant.

## Results

### Literature search and characteristics of studies included

**Fig. 1.**
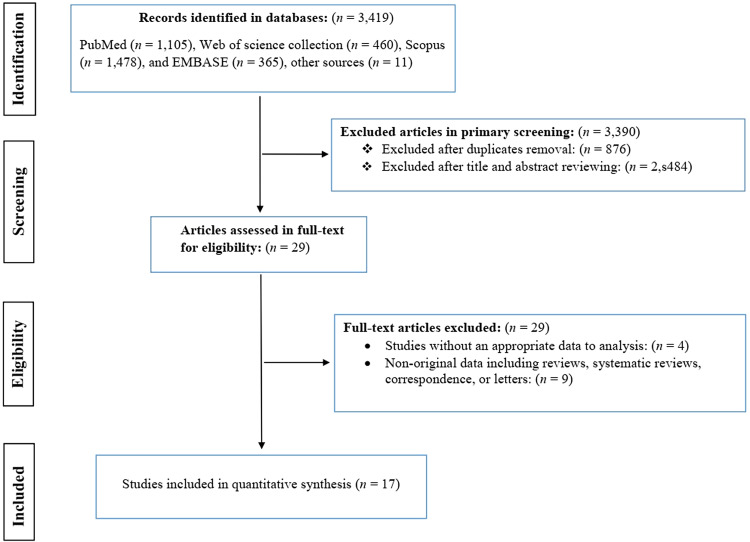
PRISMA flow chart showing study selection process.

The primary databases screening resulted in 3419 potentially relevant publications, of which 3390 were excluded after duplicate removal and the titles and abstracts screenings. After the in-depth screening of 29 full-text articles for their eligibility, 17 studies containing 24 datasets remained for the meta-analysis (Versteeg *et al*., 2003; Carter *et al*., 2004; Ngoungou *et al*., 2006*a*, 2006*b*; Idro *et al*., 2008; Opoka *et al*., 2009; Birbeck *et al*., 2010; Postels *et al*., 2012; Ngugi *et al*., 2013; Kamuyu *et al*., 2014; Wagner *et al*., 2014; Ae-Ngibise *et al*., 2015; Bistervels *et al*., 2016; Kakooza-Mwesige *et al*., 2017; Thierry *et al*., 2020; Gumisiriza *et al*., 2021; Dolo *et al*., 2022) (Fig. 1). Studies were published between 2003 and 2022. Overall, these studies included 6285 cases and 13 909 healthy controls. Eligible studies were performed in 7 countries and all in Africa (Kenya, Malawi, Mali, Tanzania, South Africa, Uganda and Ghana). Eighteen datasets, defined as epilepsy-based studies, recruited epileptic patients (2191 participants) as cases and individuals without epilepsy (2753 participants) as controls and assessed malaria infection in these subjects; while 6 datasets, defined as malaria-based studies, evaluated the prevalence or incidence of epilepsy in participants with (655 participants) and without (1075 participants) malaria infection. Epilepsy-based datasets had cross-sectional (*n* = 10) and case–control (*n* = 8) study designs, while all malaria-based studies had cohort study designs (4 prospective and 2 retrospective). Participants in 12 and 3 datasets were only children and only adults, respectively, while in 9 datasets, both children and adults participated. Considering diagnostic methods, 11 datasets used serological methods, 11 used blood smear parasitology methods and 2 used medical history for severe malaria. According to NOS, the overall quality of datasets was high for half of the datasets (*n* = 12) and moderate for the other half (Table 1). The main characteristics of the included studies are presented in Table 1.

**Table 1.** Main characteristics of included studies evaluating the relationship between malaria infection and epilepsy

Author	Country	Type of study	Type of population	Diagnostic methods	Number of cases	Positive outcome	Number of controls	Positive outcome	Quality score
Versteeg *et al*. (2003)^a^	Kenya	Retrospective cohort	Children	Slide-positive malaria	35	11	46	3	Moderate
Carter *et al*. (2004)^a^	Kenya	Retrospective cohort	Children	Slide-positive malaria	152	14	179	4	High
Ngoungou *et al*. (2006*a*)^a^	Mali	Prospective cohort	Children	Slide-positive malaria	101	5	222	0	High
Ngoungou *et al*. (2006*b*)^b^	Gabon	Case–control	Adult and children	Malaria IgG + ve (schizont)	296	45	296	12	Moderate
Idro *et al*. (2008)^b^	Kenya	Cross-sectional	Children	Slide-positive malaria	900	479	4021	482	High
Opoka *et al*. (2009)^a^	Uganda	Prospective cohort	Children	Slide-positive malaria	68	8	92	4	Moderate
Birbeck *et al*. (2010)^a^	Malawi	Prospective cohort	Children	Slide-positive malaria	132	12	264	0	High
Postels *et al*. (2012)^a^	Malawi	Prospective cohort	Children	Slide-positive malaria	167	18	272	0	High
Ngugi *et al*. (2013)^b^	Sub-Saharan Africa	Cross-sectional	Adult	Malaria IgG + ve (schizont)	563	473	703	596	Moderate
Ngugi *et al*. (2013)^b^	Sub-Saharan Africa	Cross-sectional	Children	Malaria IgG + ve (schizont)	423	333	610	491	Moderate
Kamuyu *et al*. (2014)^b^	South Africa	Case–control	Adult and children	Malaria IgG + ve (schizont)	175	40	211	70	High
Kamuyu *et al*. (2014)^b^	Tanzania	Case–control	Adult and children	Malaria IgG + ve (schizont)	278	270	345	323	High
Kamuyu *et al*. (2014)^b^	Uganda	Case–control	Adult and children	Malaria IgG + ve (schizont)	84	84	199	198	High
Kamuyu *et al*. (2014)^b^	Ghana	Case–control	Adult and children	Malaria IgG + ve (schizont)	173	173	292	291	Moderate
Kamuyu *et al*. (2014)^b^	Kenya	Case–control	Adult and children	Malaria IgG + ve (schizont)	276	209	266	203	Moderate
Wagner *et al*. (2014)^b^	South Africa	Cross-sectional	Adult and children	Malaria IgG + ve (schizont)	292	71	260	78	Moderate
Ae-Ngibise *et al*. (2015)^b^	Ghana	Cross-sectional	Adult	Malaria IgG + ve (schizont)	188	187	175	175	High
Ae-Ngibise *et al*. (2015)^b^	Ghana	Cross-sectional	Children	Malaria IgG + ve (schizont)	93	93	117	116	High
Bistervels *et al*. (2016)^b^	Kenya	Cross-sectional	Adult and children	Slide-positive malaria	150	96	2841	1679	High
Kakooza-Mwesige *et al*. (2017)^b^	Uganda	Cross-sectional	Adult	Slide-positive malaria	65	5	100	4	Moderate
Kakooza-Mwesige *et al*. (2017)^b^	Uganda	Cross-sectional	Children	Slide-positive malaria	157	7	172	10	Moderate
Thierry *et al*. (2020)^b^	Benin	Case–control	Children	Slide-positive malaria	41	29	82	12	Moderate
Gumisiriza *et al*. (2021)^b^	Uganda	Case–control	Children	Medical history	154	38	153	43	Moderate
Dolo *et al*. (2022)^b^	Mali	Cross-sectional	Adult and children	Medical history	1322	470	1991	88	High

a Malaria-based studies.

b Epilepsy-based studies.

### Overall analysis and subgroup analyses

**Fig. 2.**
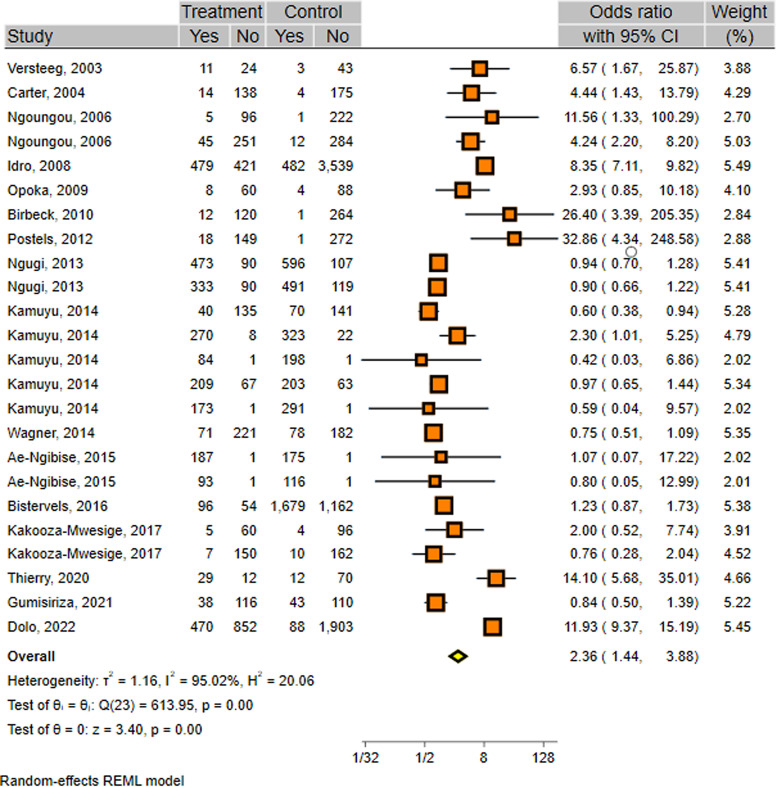
Forest plot, pooled with random effects, regarding the association between malaria infection and epilepsy.

As displayed in Table 1 and Fig. 2, the overall meta-analysis indicated a significant positive association between malaria infection and epilepsy development (OR 2.36; 95% CI 1.44–3.88) (Table 2 and Fig. 2). In subgroup analysis, malaria-based studies showed a strong positive association (OR 7.10; 95% CI 3.50–14.38), while this association was marginally significant in epilepsy-based studies (OR 1.70; 95% CI 0.99–2.91). Considering the study design, both retrospective (OR 5.2; 95% CI 2.17–12.46) and prospective (OR 10.87; 95% CI 3.06–38.58) cohorts indicated significant positive associations, while cross-sectional (OR 1.74; 95% CI 0.84–3.62) and case–control (OR 1.64; 95% CI 0.71–3.79) studies showed non-significant positive associations (Table 2). Considering the diagnostic methods, studies that had used the blood smear method yielded a significant positive association (OR 4.80; 95% CI 2.36–9.77), while other methods showed non-significant positive associations. Regarding the type of participants, quality of studies and year of publications, studies that used only children (OR 3.92; 95% CI 1.81–8.50), those with high qualities (OR 3.61; 95% CI 1.62–8.04) and those published before 2010 (OR 6.39; 95% CI 4.25–9.62) showed significant positive associations. More details on subgroup analyses are presented in Table 2. The funnel plot demonstrated no publication bias in the studies included in this meta-analysis (Fig. S2).

**Table 2.** Sub-group analysis of the pooled prevalence and odds ratios for the association between malaria and epilepsy

Variables	Datasets (*n*)	Pooled prevalence of outcome in cases % (95% CI)	Pooled prevalence of outcome in controls % (95% CI)	Odds ratios (95% CI)	Heterogeneity (*I*^2^%)
Study design					
Epilepsy-based	18	63.47 (46.43–78.96)	54.26 (33.92–73.90)	1.70 (0.99–2.91)	95.79
Malaria-based	6	10.70 (6.74–15.40)	1.28 (0.22–2.95)	7.10 (3.50–14.38)	20.25
Type of studies					
Prospective cohort	4	8.96 (6.27–12.06)	0.75 (0.03–2.07)	10.87 (3.06–38.58)	48.16
Retrospective cohort	2	12.25 (7.79–17.49)	2.69 (0.79–5.43)	5.2 (2.17–12.46)	0
Cross-sectional	10	58.15 (37.62–77.32)	49.03 (24.50–73.80)	1.74 (0.84–3.62)	97.19
Case–control	8	69.98 (36.66–94.52)	60.74 (25.63–90.49)	1.64 (0.71–3.79)	90.88
Diagnostic method					
Malaria IgG + ve (schizont)	11	80.02 (57.97–95.26)	79.04 (57.01–94.60)	1.08 (0.71–1.65)	80.33
Slide-positive malaria	11	21.84 (8.48–39.08)	6.85 (0.09–21.31)	4.80 (2.36–9.77)	89.99
Medical history	2	34.34 (31.93–36.79)	5.37 (4.44–6.38)	3.19 (0.23–43.09)	98.83
Type of population					
Adult and children	9	65.44 (39.43–87.31)	58.65 (27.97–86.02)	1.59 (0.75–3.37)	95.11
Adult	3	69.25 (22.70–99.20)	68.42 (14.33–99.99)	0.97 (0.72–1.31)	0
Children	12	32.58 (14.43–53.88)	16.26 (3.39–35.81)	3.92 (1.81–8.50)	93.66
Quality score					
Moderate	12	44.37 (22.23–67.74)	34.66 (12.64–60.83)	1.64 (0.93–2.90)	91.11
High	12	54.72 (32.61–75.91)	40.04 (18.12–64.29)	3.61 (1.62–8.04)	95.86
Years					
2003–2010	7	17.35 (4.15–36.63)	3.43 (0.38–8.83)	6.39 (4.25–9.62)	33.34
2010–2022	17	63.72 (44.45–80.97)	56.28 (34.01–77.32)	1.58 (0.89–2.81)	95.42
Total	24	49.57 (34.20–64.99)	37.35 (21.05–55.26)	2.36 (1.44–3.88)	95.02

Although the overall heterogeneity was high (*I*^2^ = 95.02%, *Q* = 613.95, *P* < 0.001), it was low or moderate in some sub-groups, especially for those indicating significant positive association, including malaria-based studies (*I*^2^ = 20.2%, *Q* = 6.53, *P* = 0.258), retrospective (*I*^2^ = 0.0%, *Q* = 0.19, *P* = 0.666), prospective cohorts (*I*^2^ = 48.1%, *Q* = 5.77, *P* = 0.123) and studies published before 2010 (*I*^2^ = 33.3%, *Q* = 8.82, *P* = 0.184) (Table 2). Multivariate meta-regression was performed to further explore the sources of heterogeneity based on the study design, type of participants, publication year and diagnostic methods. Multivariate meta-regression analysis indicated that only the diagnostic method (*C* = −0.0001; *P* < 0.001) could be the source of heterogeneity and accounted for 64.5% of between-study heterogeneity.

### Sensitivity and cumulative analysis

**Fig. 3.**
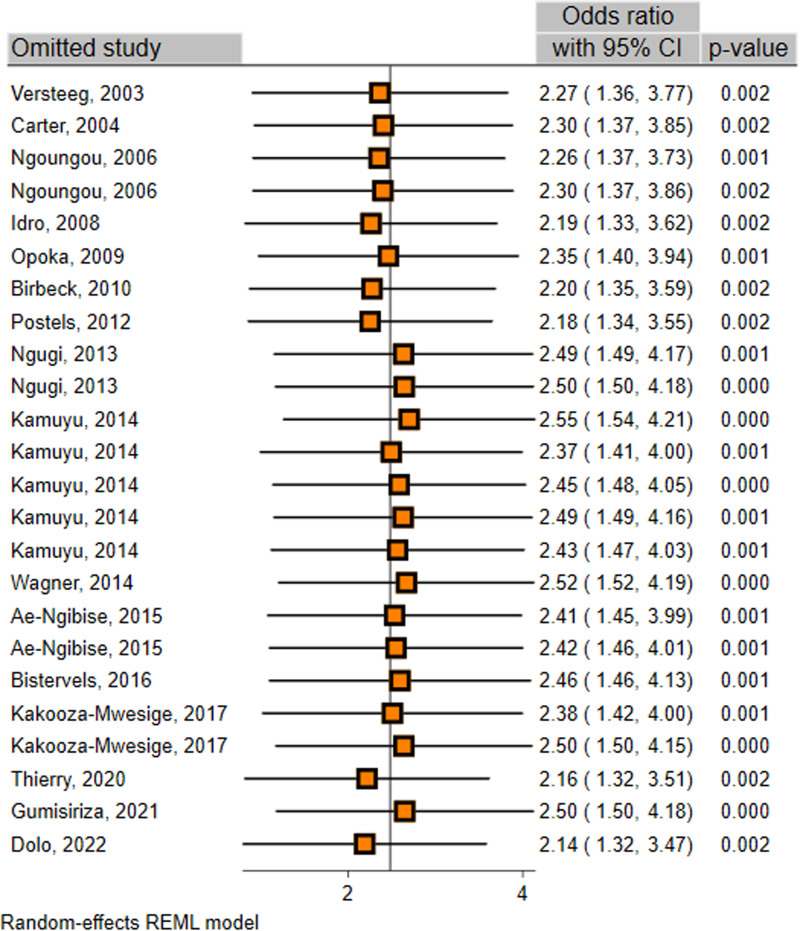
Sensitivity analysis after each study was removed.

The sensitivity analysis was conducted to evaluate the possible influence of any individual study on the main results. The analysis assessed whether omitting 1 study substantially altered the main outcome or magnitude of the summary estimates of the remainders. This analysis indicated that the exclusion of any individual study did not significantly alter the overall results of the meta-analysis (Fig. 3), implying high stability of the results.

**Fig. 4.**
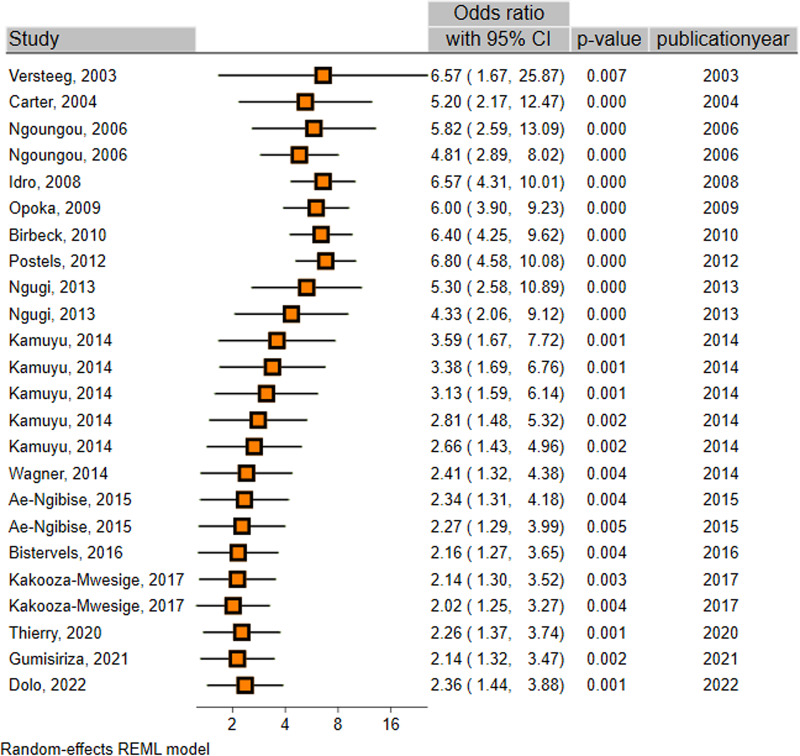
Cumulative meta-analysis regarding the association between malaria infection and epilepsy.

A cumulative meta-analysis was also performed to evaluate the consistency of the evidence over the years as recent studies were added. The cumulative analysis indicated that between 2003 and 2022, there was a positive association between malaria infection and epilepsy development but with a swinging effect size and narrowing CIs (Fig. 4).

## Discussion

Epilepsy is a highly prevalent neurological disorder in LMIC, especially in malaria-endemic areas; however, a definitive causative relationship is yet to be established. The results of this systematic review and meta-analysis are consistent with a previous study (Christensen and Eslick, 2015), indicating a significant positive association between malaria infection and epilepsy, particularly for patients who survived CM. All subgroup analyses also showed a positive association, although the results were more significant in cohort studies that followed patients with CM. Moreover, the epilepsy-based studies indicated a marginally significant association, suggesting future well-designed studies are required to establish a definitive association between malaria infection and epilepsy development.

The association between malaria and epilepsy development is likely explained by more than 1 mechanism, possibly due to CM and its related inflammation (Singh *et al*., 2020). However, the pathophysiological mechanisms leading to CM and its related neurological complications are not yet fully understood (Christensen and Eslick, 2015). The current knowledge is largely based on a few reports of human autopsy and findings from models of *Plasmodium berghei* infection in C57BL/6J mice (Oca *et al*., 2013; Singh *et al*., 2020). The key processes that were elucidated in these studies were cytoadherence of the parasitized erythrocytes and inflammation. It is demonstrated that upregulation of infections-induced inflammatory markers such as tumour necrosis factor, intracellular adhesion mediator 1 and angiopoietin 2 can induce endothelial damage in the cerebral blood vessels (Conroy *et al*., 2012; Storm and Craig, 2014; Cruz *et al*., 2016; O'Regan *et al*., 2016; Shabani *et al*., 2017; Storm *et al*., 2019) and maybe effective in the initiation of seizures and other status epilepticus. Moreover, it is suggested that vascular-ischaemic lesions resulting from the sequestration of parasitized erythrocytes and the Durck's malarial granuloma that comprises of reactive astrocytes during the acute attack of CM could lead to structural damage to the brain and induce epileptogenic lesions (Aleem, 2005; Ngoungou and Preux, 2008). Finally, neurotoxins such as quinolinic acid and autoantibodies to voltage-gated calcium channels that are induced in CM might have a role in the developing of seizures in children with severe malaria (Dobbie *et al*., 2000; Lang *et al*., 2005; Ngoungou and Preux, 2008), which requires further investigations.

Although the present study comprehensively evaluated the associations between malaria infection and epilepsy, some limitations should be acknowledged in the interpretation of our findings. First, some studies used medical history or serological methods to define malaria, which could be subject to diagnosis biases in pooled OR; however, subgroup analysis was performed in this study according to diagnostic criteria to overcome this limitation. Second, there were insufficient data considering sex, age or other comorbidities, and notably, information about other infections was underrepresented in some included studies. Despite these drawbacks, the findings and interpretations presented here provided useful insights concerning the association between malaria infection and epilepsy.

In conclusion, our findings indicated that patients with malaria, especially those with CM, are at a higher risk of epilepsy development; however, several gaps remain in our understanding of pathophysiological mechanisms of this event. Therefore, further human or experimental studies are needed to focus on long-term sequelae of CM such as epilepsy; neuropathological and physiological changes leading to these sequelae; the impact of therapeutic agents that are used during CM episodes; and the impact of other epileptogenic intracranial infections on the development of epilepsy and other neurological sequelae.

## Data Availability

Data supporting results are provided within the article and in the Supplementary materials.

## Supplementary material

For supplementary material accompanying this paper visit https://doi.org/10.1017/S0031182022001780. click here to view supplementary material
