# Supplementary figures and images for: Malaria infection and the risk of epilepsy: a meta-analysis
Source: Parasitology. 2023 Jan 27;150(4):382–90. doi: 10.1017/S0031182022001780 (PMC10090621; doi:10.1017/S0031182022001780)

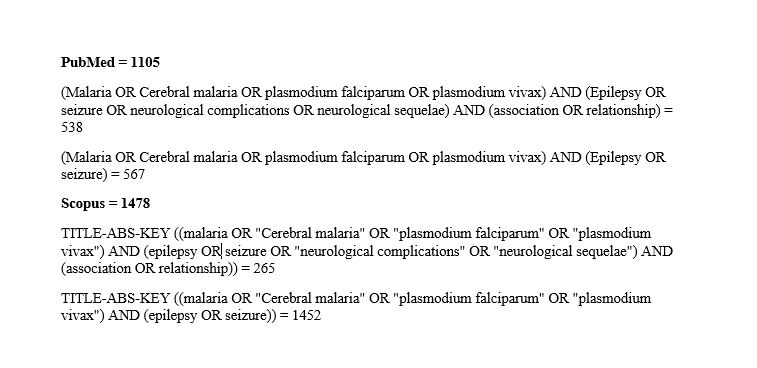

Supplement: Supplementary file 1 [file S0031182022001780sup.zip › S0031182022001780sup001.jpg]

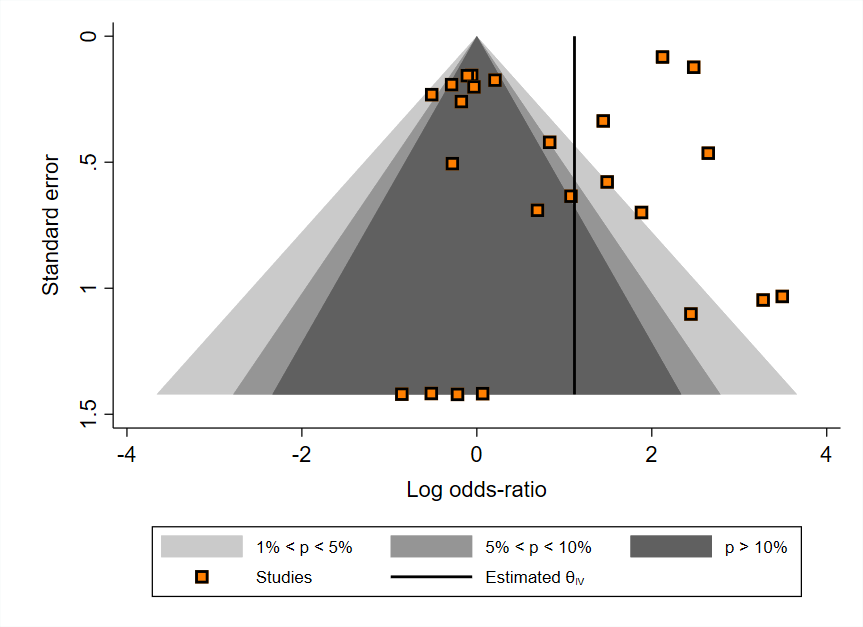

Supplement: Supplementary file 1 [file S0031182022001780sup.zip › S0031182022001780sup002.tif]
